# Supplementary material for: Higher tree diversity increases soil microbial resistance to drought
Source: Commun Biol. 2020 Jul 14;3:377. doi: 10.1038/s42003-020-1112-0 (PMC7360603; doi:10.1038/s42003-020-1112-0)
Supplement: Supplementary file 1 — Supplementary Information [file 42003_2020_1112_MOESM1_ESM.pdf]

Higher tree diversity increases soil microbial resistance to drought – Supplementary Materials

Supplementary Figures

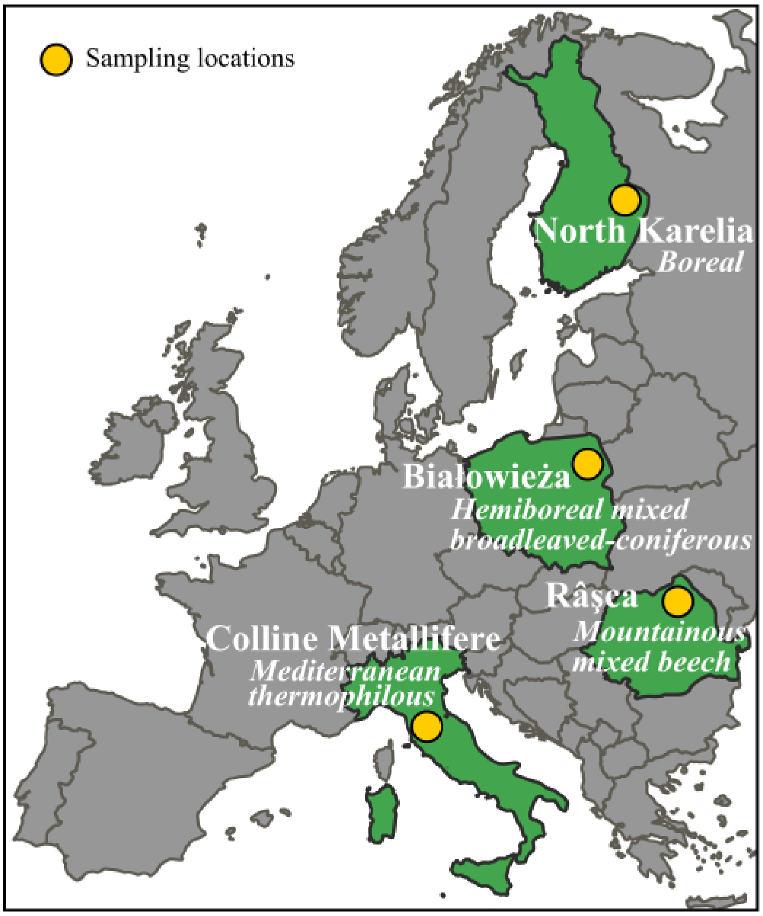

Supplementary Figure 1. Soil was sampled from four forest types at four sites located in four European countries: Colline Metallifere, Italy (Mediterranean thermophilous); Râşca, Romania (mountainous mixed beech); Białowieża, Poland (hemiboreal mixed broadleaved-coniferous); and North Karelia, Finland (boreal). Sampling was during phenological spring when deciduous trees were leafing out at all four sites (end of April in Italy, early May in Poland, late May in Romania, and mid-June in Finland).

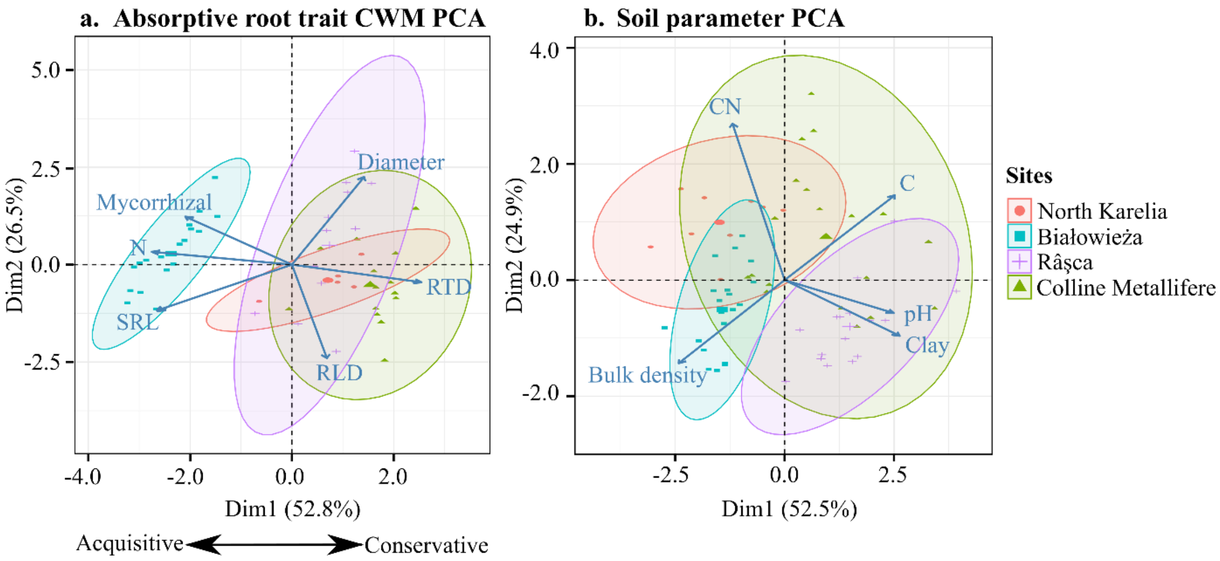

Supplementary Figure 2. PCA ordination of **a.** absorptive root trait CWM and **b.** soil parameters. Abbreviations: Absorptive root traits: SRL= Specific root length (m g^{-1}), Diam= Root diameter (mm), TD= Root tissue density (g cm^{-3}), LD= Root length density (cm cm^{-3}), Mycorrhizal= Ectomycorrhizal colonization intensity (number cm^{-1}), N= Nitrogen content (%). Soil parameters (for the first 10 cm of the A horizon): C= Carbon content (mg g^{-1} soil), CN= Carbon to Nitrogen ratio, pH= pH ($CaCl_2$), Clay= Clay content (%), BD= Bulk density (g cm^{-3}).

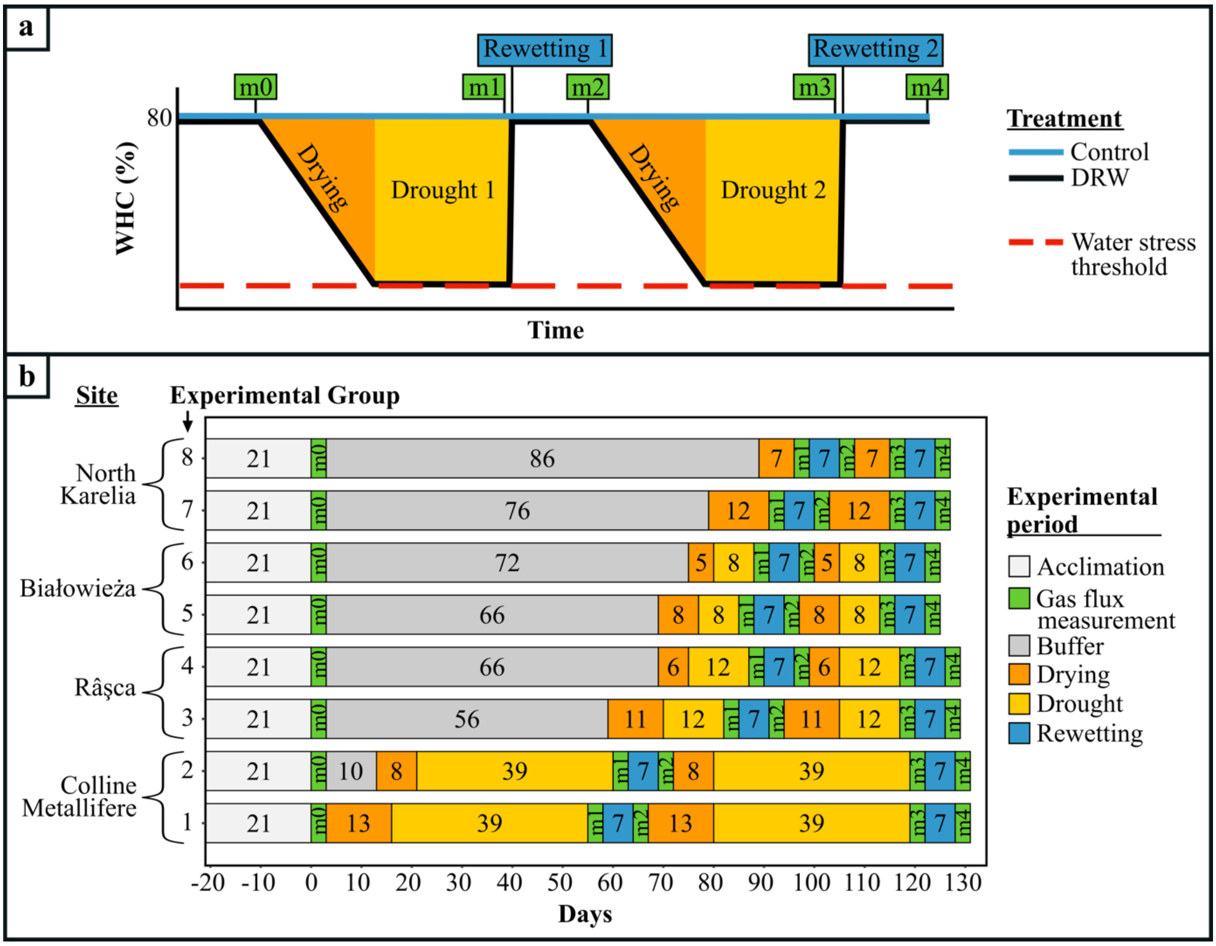

Supplementary Figure 3. **a.** Schema of the two drying-rewetting (DRW) cycles during the experiment for the DRW treatment (black line) and the control treatment (blue line), which were kept at 80% water holding capacity (WHC) throughout the experiment. **b.** The microcosms were grouped into eight experimental groups by soil drying speed (i.e. the time needed to reach the threshold soil water potential) to synchronize the end of the second DRW cycle. The color indicates the different experimental periods of the DRW cycles and the corresponding duration is indicated (in days). The gas flux measurements were taken before the DRW treatment started (m0), after the first drought (m1), after the rewetting (m2), after the second drought (m3), and after the second rewetting period (m4). The final measurements had to be spread out over several days as it was logistically impossible to measure 384 microcosms at the same time.

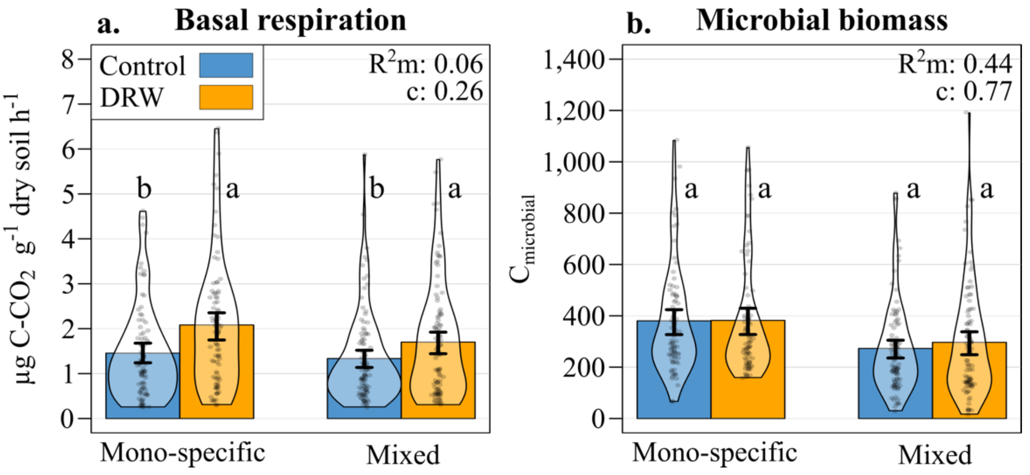

Supplementary Figure 4. CO_2 basal respiration (µg C-CO_2 g^{-1} dry soil h^{-1}) and microbial biomass ($C_{microbial}$) after two drying and rewetting cycles (Rewetting 2; Supplementary Fig. 3) for the control (blue) and DRW (yellow) treatments on soil from either mono-specific or 3-species mixed stands. The most parsimonious model R^2 (marginal and conditional), standard error bars, and the significant differences between the control treatment, DRW treatment, mono-specific stands, and mixed stands, indicated by lower-case letters, are from two GLMMs run on CO_2 basal respiration and microbial biomass (Supplementary Table 4).

Supplementary Tables

Supplementary Table 1. Description of the four sample sites, including the tree species pool, and site-specific plot design. Soil measurements are from the top 10 cm of soil.

	**North Karelia**	**Białowieża**	**Râşca**	**Colline Metallifere**
Country	Finland	Poland	Romania	Italy
Latitude/longitude (°)	62.6, 29.9	52.7, 23.9	47.3, 26.0	43.2, 11.2
Ownership	State, large private forest companies	State	State	State
MAT, MAP[a]	2.1 ◦C, 700 mm	6.9 ◦C, 627 mm	6.8 ◦C, 800 mm	13 ◦C, 850 mm
Topography, altitude[b]	Flat, 80–200 m	Flat, 135–185 m	Medium-steep slopes, 600–1000 m	Medium-steep slopes, 260–525 m
Study area (km x km)	150 × 150	30 × 40	5 × 5	50 × 50
Forest type[c]	Boreal	Hemiboreal, nemoral coniferous, mixed broadleaved-coniferous	Mountainous mixed beech	Thermophilous deciduous
Basal area (m^2 ha^{-1}) [I]	233.6	392.9	501.3	272.6
Soil type [II]	Podzol or entic podzol	Luvisols cambisol	Eutric cambisol	Cambisol
Sand, silt, clay [I]	48%, 47%, 5%	65%, 29%, 6%	13%, 60%, 27%	17%, 65%, 18%
Soil C; N mg g^{-1} soil [II]	37.8; 1.7	28.4; 1.7	49.2; 3.5	50.4; 2.6
Soil pH [II]	3.9	3.8	4.6	4.6
Sampling date (yyyy/mm/dd)	2017/06/12-16	2017/05/05-10	2017/05/22-28	2017/04/10-16
Target tree species	*Betula sp.*<br>*Picea abies*<br>*Pinus sylvestris*	*Betula sp.*<br>*Carpinus betulus*<br>*Picea abies*<br>*Pinus sylvestris*<br>*Quercus robur*	*Abies alba*<br>*Acer pseudoplatanus*<br>*Fagus sylvatica*<br>*Picea abies*	*Castanea sativa*<br>*Ostrya carpinifolia*<br>*Quercus cerris*<br>*Quercus ilex*<br>*Quercus petraea*
Number of mono-specific stand plots	6	6	8	10
Number of mixed stand plots	3	14	8	9

[a] MAT: mean annual temperature, MAP: mean annual precipitation.
[b] Altitude in meters above sea level.
[c] Categories of the European Environment Agency[1].
[I] Data from SoilForEUROPE project
[II] Data from FunDivEUROPE project

Reference

1. EEA. *European Forest Types. Categories and types for sustainable forest management reporting and policy, 2nd ed. EEA Technical Report 09/2006.* (2007). doi:10.3832/efor0425-003

Supplementary Table 2. List of the variables, and mean values plus or minus the standard deviation, used for the soil parameter PCA and for the absorptive root community weighted mean (CWM) and functional dispersion (FDis) calculations for each site and for mono-specific (mono) and mixed stands. The first axis scores for the soil parameter and root CWM are also included, as well as the root FDis values.

Variable	Units	Finland		Poland		Romania		Italy	
		Mono	Mixed	Mono	Mixed	Mono	Mixed	Mono	Mixed
Soil Parameters									
Bulk density	g cm^{-3}	1.03 ± 0.11	1.02 ± 0.04	1.04 ± 0.09	1.01 ± 0.07	0.92 ± 0.04	0.93 ± 0.08	0.9 ± 0.07	0.87 ± 0.07
C	mg g^{-1}	37.91 ± 14.45	37.59 ± 3.03	29.41 ± 7.44	27.97 ± 4.43	51.69 ± 18.02	46.67 ± 14.53	49.9 ± 15.8	50.89 ± 11.12
Clay	%	5.23 ± 0.74	4.97 ± 1.06	5.85 ± 2.3	5.77 ± 1.29	27.43 ± 7.62	27.44 ± 4.66	16.86 ± 5	19.43 ± 5.88
C:N	ratio	24.64 ± 3.76	21.97 ± 4.01	17.27 ± 2.81	16.49 ± 2.02	13.88 ± 1.41	13.91 ± 0.97	19.63 ± 2.9	20.3 ± 4.01
pH		3.98 ± 0.25	3.66 ± 0.19	3.66 ± 0.31	3.81 ± 0.24	4.81 ± 0.54	4.44 ± 0.74	4.59 ± 1.17	4.71 ± 0.97
Absorptive root traits									
Diameter	mm	0.45 ± 0.24	0.35 ± 0	0.38 ± 0.06	0.3 ± 0.03	0.38 ± 0.04	0.37 ± 0.05	0.49 ± 0.04	0.42 ± 0.04
ECM colonization	number cm^{-1}	1.4 ± 0.82	3.04 ± 1.76	1.71 ± 0.78	2.09 ± 0.53	1.58 ± 1.2	2.1 ± 0.74	1.27 ± 0.69	1.72 ± 0.78
N	%	0.99 ± 0.12	3.61 ± 0.17	1.66 ± 0.21	5.67 ± 0.41	1.4 ± 0.16	4.26 ± 0.52	1.01 ± 0.17	2.25 ± 0.55
RLD	cm cm^{-3}	1.74 ± 0.37	2.09 ± 0.39	2.06 ± 0.45	3.65 ± 0.84	1.93 ± 0.56	1.91 ± 1.32	1.55 ± 0.55	3.48 ± 0.84
RTD	g cm^{-3}	0.99 ± 0.13	3.61 ± 0.2	1.66 ± 0.23	5.67 ± 0.42	1.34 ± 0.11	4.26 ± 0.55	1.01 ± 0.17	2.25 ± 0.58
SRL	m g^{-1}	0.41 ± 0.07	0.36 ± 0.01	0.4 ± 0.07	0.32 ± 0.03	0.51 ± 0.12	0.39 ± 0.05	0.38 ± 0.06	0.34 ± 0.05
Surface area	cm^2	22.8 ± 8.06	27.46 ± 3.32	25.07 ± 7.43	47.4 ± 10.04	15.91 ± 8.2	26.14 ± 7.84	19.84 ± 6.39	31.59 ± 5.21
Soil Parameter PC1		-1.47 ± 1.12	-1.52 ± 0.25	-1.59 ± 0.59	-1.35 ± 0.34	1.71 ± 0.51	1.28 ± 1.12	0.78 ± 1.51	1.13 ± 0.79
Absorptive root CWM PC1		0.65 ± 0.93	0.84 ± 0.1	-2.15 ± 0.69	-2.47 ± 0.49	0.74 ± 0.89	0.74 ± 0.36	1.36 ± 0.95	1.74 ± 0.23
Absorptive root FDis		0 ± 0	1.75 ± 0.13	0 ± 0	1.47 ± 0.37	0 ± 0	1.75 ± 0.25	0 ± 0	1.67 ± 0.35

Supplementary Table 3. The most parsimonious model results testing the resistance-recovery correlation for CO_2 and N_2O in relation to tree species number: R^2 marginal (R^2m), and R^2 conditional (R^2c), estimated slope (Est.), standard error (SE), degree of freedom (df), t-value, and p-values. Red and blue estimate values indicate positive and negative relationships, respectively. Explanatory variables are abbreviated as: tree species number (Sp.No.), recovery and tree species number interaction (Recovery:Sp.No.), the change between the first and second DRW cycle (Cycle), and tree species number and cycle interaction (Sp.No.:Cycle). Dashes indicate explanatory variables not retained in the most parsimonious model and p-values are coded as such: 0.1> and <0.05 '.'; 0.05> and <0.01 '*'; 0.01> and <0.001 '**', 0.001> '***'.

	**CO2 flux recovery**						**N2O flux recovery**					
	**R^2m =**	**0.012**	**R^2c =**	**0.173**	**AIC= 959.3**		**R^2m =**	**0.071**	**R^2c =**	**0.545**	**AIC= 460.7**	
	**Est.**	**SE**	**df**	**t-value**	**p-value**		**Est.**	**SE**	**df**	**t-value**	**p-value**	
Resistance	-	-	-	-	-		0.78	0.14	321.70	5.38	1.4E-07	***
Sp.No.	-	-	-	-	-		-	-	-	-	-	
Res.:Sp.No.	-	-	-	-	-		-	-	-	-	-	
Cycle 2	-0.22	0.10	297.60	-2.25	2.6E-02	*	0.14	0.07	278.97	1.93	0.05	.
Res.:Cycle2	-	-	-	-	-		-0.55	0.14	291.33	-3.89	0.00	***
Sp.No.:Cycle	-	-	-	-	-		-	-	-	-	-	

Supplementary Table 4. The most parsimonious model results: R^2 marginal (R^2m), and R^2 conditional (R^2c), estimated slope (Est.), standard error (SE), degree of freedom (df), t-value, and p-values for the basal respiration and microbial biomass following two DRW cycles. Red and blue estimate values indicate positive and negative relationships, respectively, based on estimate values not significance. Explanatory variables are abbreviated as: DRW treatment (DRW), tree species number (Sp.No.), tree species number and DRW treatment interaction (Sp.No.:DRW), topsoil properties (Soil parameters), absorptive root functional dispersion (Root FDis), absorptive root community weighted mean traits (Root CWM). Dashes indicate explanatory variables not retained in the most parsimonious model, p-values are coded as such: 0.1> and <0.05 ‘.’; 0.05> and <0.01 ‘*’; 0.01> and <0.001 ‘**’, 0.001> ‘***’, variables were sometimes retained but not significant.

	**Basal respiration**						**Microbial biomass**					
	**R^2m =**	**0.057**	**R^2c =**	**0.258**	**AIC= 1106.8**		**R^2m =**	**0.444**	**R^2c =**	**0.771**	**AIC= -543.5**	
	**Est.**	**SE**	**df**	**t-value**	**p-value**		**Est.**	**SE**	**df**	**t-value**	**p-value**	
DRW	0.40	0.13	5.7	3.18	0.02	*	-	-	-	-	-	
Sp.No.	-	-	-	-	-		-0.06	0.03	59.18	-1.86	0.07	.
Sp.No.:DRW	-	-	-	-	-		-	-	-	-	-	
Soil parameters	-0.09	0.05	63.6	-1.72	9.1E-02	.	-	-	-	-	-	
Root FDis	-	-	-	-	-		-	-	-	-	-	
Root CWM	0.10	0.05	64.0	1.98	5.2E-02	.	0.07	0.01	59.1	7.64	2.2E-10	***
