# Supplementary material for: Higher tree diversity increases soil microbial resistance to drought
Source: Commun Biol. 2020 Jul 14;3:377. doi: 10.1038/s42003-020-1112-0 (PMC7360603; doi:10.1038/s42003-020-1112-0)
Supplement: Supplementary file 2 — Description of Additional Supplementary Files [file 42003_2020_1112_MOESM2_ESM.pdf]

Description of Additional Supplementary Files

File Name: **Supplementary Data 1**

Description: Plot and tree information and response variable data: CO_2 and N_2O fluxes (µl C-CO_2 or N-N_2O g^{-1}dry soil h^{-1}), cumulative CO_2 and N_2O fluxes (µl C-CO_2 or N-N_2O g^{-1} dry soil h^{-1}), dissolved organic carbon (DOC; mg C kg^{-1} dry soil), total dissolved nitrogen (TDN; mg N kg^{-1} dry soil), metabolic quotient (qCO_2; µg C-CO_2 mg^{-1} Cmicrobial h-1), and CO_2 and N_2O flux resistance and recovery indices.
