# Supplementary material for: Higher tree diversity increases soil microbial resistance to drought
Source: Commun Biol. 2020 Jul 14;3:377. doi: 10.1038/s42003-020-1112-0 (PMC7360603; doi:10.1038/s42003-020-1112-0)
Supplement: Supplementary file 4 — Reporting Summary [file 42003_2020_1112_MOESM4_ESM.pdf]

nature research

Corresponding author(s): Lauren Gillespie

Last updated by author(s): Jun 4, 2020

# Reporting Summary

Nature Research wishes to improve the reproducibility of the work that we publish. This form provides structure for consistency and transparency in reporting. For further information on Nature Research policies, see Authors & Referees and the Editorial Policy Checklist.

## Statistics

For all statistical analyses, confirm that the following items are present in the figure legend, table legend, main text, or Methods section.

| n/a | Confirmed | |
|---|---|---|
| ☐ | ☒ | The exact sample size (*n*) for each experimental group/condition, given as a discrete number and unit of measurement |
| ☐ | ☒ | A statement on whether measurements were taken from distinct samples or whether the same sample was measured repeatedly |
| ☐ | ☒ | The statistical test(s) used AND whether they are one- or two-sided<br>*Only common tests should be described solely by name; describe more complex techniques in the Methods section.* |
| ☐ | ☒ | A description of all covariates tested |
| ☐ | ☒ | A description of any assumptions or corrections, such as tests of normality and adjustment for multiple comparisons |
| ☐ | ☒ | A full description of the statistical parameters including central tendency (e.g. means) or other basic estimates (e.g. regression coefficient) AND variation (e.g. standard deviation) or associated estimates of uncertainty (e.g. confidence intervals) |
| ☐ | ☒ | For null hypothesis testing, the test statistic (e.g. *F*, *t*, *r*) with confidence intervals, effect sizes, degrees of freedom and *P* value noted<br>*Give P values as exact values whenever suitable.* |
| ☒ | ☐ | For Bayesian analysis, information on the choice of priors and Markov chain Monte Carlo settings |
| ☐ | ☒ | For hierarchical and complex designs, identification of the appropriate level for tests and full reporting of outcomes |
| ☒ | ☐ | Estimates of effect sizes (e.g. Cohen's *d*, Pearson's *r*), indicating how they were calculated |

*Our web collection on statistics for biologists contains articles on many of the points above.*

## Software and code

Policy information about availability of computer code

| | |
|---|---|
| Data collection | *No code was used for data collection.* |
| Data analysis | *The R software (version 3.5.3) was used for all statistical analyses and figures using the 'dredge' function in the MuMIn package (version 1.43.6) for the GLMs and the 'pirateplot' function in the YaRrr! package (version 0.1.5) for the figures. For FDis and CWM calculations we used the R function 'dbFD' in the FD package (version 1.0-12).* |

For manuscripts utilizing custom algorithms or software that are central to the research but not yet described in published literature, software must be made available to editors/reviewers. We strongly encourage code deposition in a community repository (e.g. GitHub). See the Nature Research guidelines for submitting code & software for further information.

## Data

Policy information about availability of data

All manuscripts must include a data availability statement. This statement should provide the following information, where applicable:

- Accession codes, unique identifiers, or web links for publicly available datasets
- A list of figures that have associated raw data
- A description of any restrictions on data availability

*The datasets generated during and/or analyzed during the current study are available from the corresponding author on reasonable request. This data is stored as excel files on a data portal associated with the FunDivEUROPE and SoilForEUROPE projects.*

# Field-specific reporting

Please select the one below that is the best fit for your research. If you are not sure, read the appropriate sections before making your selection.

☐ Life sciences ☐ Behavioural & social sciences ☒ Ecological, evolutionary & environmental sciences

For a reference copy of the document with all sections, see nature.com/documents/nr-reporting-summary-flat.pdf

# Life sciences study design

All studies must disclose on these points even when the disclosure is negative.

| | |
|---|---|
| Sample size | *not applicable* |
| Data exclusions | *not applicable* |
| Replication | *not applicable* |
| Randomization | *not applicable* |
| Blinding | *not applicable* |

# Behavioural & social sciences study design

All studies must disclose on these points even when the disclosure is negative.

| | |
|---|---|
| Study description | *not applicable* |
| Research sample | *not applicable* |
| Sampling strategy | *not applicable* |
| Data collection | *not applicable* |
| Timing | *not applicable* |
| Data exclusions | *not applicable* |
| Non-participation | *not applicable* |
| Randomization | *not applicable* |

# Ecological, evolutionary & environmental sciences study design

All studies must disclose on these points even when the disclosure is negative.

| | |
|---|---|
| Study description | This soil microcosm experiment in growth chambers explored the effects of drying and rewetting (DRW) cycles on soil microbial functioning in soil sampled from four sites along a latitudinal gradient in Europe and whether the tree species diversity of the sampled plot (one versus three species) influenced this effect. Half of the soil microcosms (n=192; three replicates for each of the 64 plots) were subjected to two drying and rewetting cycles (drought severity and duration were site-specific) and half (n=192) were kept at optimum conditions as controls. Gas flux measurements (CO2 and N2O) were conducted at key points during the experiment (before drought, after drought, and after rewetting) for both of the DRW cycles, measuring soil microbial respiration and denitrification activities to estimate microbial resistance and recovery to DRW cycles. At the end of the experiment, further analyses were run to explore microbial stress levels (qCO2) and potential nutrient leaching (DOC and TDN). We then compared results between treatments and between soil from one tree species plots and three tree species plots. |
| Research sample | *Topsoil samples were collected from forest plots at each site (described below) that had either mono-specific tree stands or 3-species mixed stands. Twenty-five soil cores (10 cm deep, 5.3 cm diameter) were collected at each of the 64 plots and passed through a 2 mm sieve. The topsoil was chosen since it is the most active soil horizon that is responsible for the majority of microbial activity and associated biogeochemical cycling, and will be most affected by climate change.* |
| Sampling strategy | The sites used in this study are part of a permanent network of existing mature forest plots across Europe established in 2011-2012 (see Baeten et al. 2013 and for detailed descriptions). We included four sites ranging over a large climatic gradient: North Karelia (Finland), Białowieża (Poland), Râşca (Romania), and Colline Metallifere (Italy), which correspond to typical boreal forests, hemiboreal mixed broadleaved-coniferous, mountainous mixed beech, and Mediterranean thermophilous, respectively. At each site, we selected |

30 m × 30 m forest plots dominated by either one tree species (mono-specific stands) or by three co-dominating tree species, hereafter referred to as mixed stands, resulting in a total of 34 species combinations (species were considered co-dominant if they composed >15% of the stand). Each site differed in total species numbers, species identity, and species combinations. There were two replicates per tree species for the mono-specific plots of each site, except for Picea abies and Quercus robur which were only replicated once and Betula pendula which had no mono-specific plot in Białowieża. There was a minimum of three mixed species plot replicates per site that were composed of any of the target species present at the site, i.e. the replicate mixed plots at each site did not necessarily have the same tree species combinations. There were 64 plots in total. The sampling design with the total plot number, their distribution over four forest ecosystems, and including a wide range of tree species is well suited to address the generality of our hypothesis that microbial responses to DRW cycles are modified by tree species mixing but poorly suited to identify site-specific patterns with plot numbers too limiting within specific sites for robust testing.
Within each plot, we selected five tree triplets, a triplet being a triangle of three tree individuals within a maximum distance of 8 m from each other and no obstructing tree individuals within the triangle. Each triplet was composed of either the same species in the mono-specific stands (mono-specific triplet) or the three tree species present in the mixed stands (mixed triplet). At the estimated tree individual size weighted (based on individual diameter at breast height) center within the triangle, we collected five soil cores from the topsoil (10 cm deep, 5.3 cm diameter) after the litter layer had been removed. The five soil cores were spaced at roughly 35 cm from each other circling the center point (approximate sampled area 50 cm × 50 cm). A depth of 10 cm was selected because it is the standard topsoil sampling depth in soil ecology, and has the highest soil microbial activity and is under the most influence from the plant community. All soil cores from each sampling location (i.e. tree triplet) within a plot were then sieved together through a 2 mm sieve and air-dried immediately after sampling for transportation and experiment preparation.

Data collection

*Soil microbial respiration and denitrification activities were estimated by measuring CO2 and N2O fluxes in the microcosms. We measured the CO2 and N2O fluxes before the first drought, at the end of the first and second drought periods, and seven days following the rewetting of the drought exposed microcosms. The second DRW cycle started immediately after the post-rewetting flux measurement. Flux measurements were done concurrently in both the DRW and control microcosms of the same group.*
*For the CO2 measurements, we sealed the microcosms for 23 hours to allow CO2 accumulation, which we then measured using a MicroGC (S-Series, SRA Intruments, Marcy l'Etoile, France). We subsequently replaced the air in the microcosms with 90% helium and 10% acetylene to prevent N2O reduction to N2 (allowing the measurement of denitrified N as N2O), then incubated the microcosms for another 23-hour period to again allow gas accumulation under anaerobic condition. The N2O concentration was then measured using a GC CP-3800 equipped with an electron capture detector (Varian, Palo Alto, USA). Flux rates were calculated as the amount in µg of CO2 or N2O produced per gram of soil per hour (µg C-CO2 or N-N2O h-1 g-1 dry soil). Data was recorded automatically by the machines used and downloaded as an excel sheet. The five gas flux measurements were used to calculate the cumulative gas flux over the experiment as well as resistance and recovery indices (following Nimmo et al., 2015).*
*After the end of experiment, the soil microbial biomass was estimated using the substrate-induced-respiration (SIR) method, which was then used to calculate the metabolic quotient (qCO2) defined as the C-CO2 respired per unit of microbial biomass (ng C-CO2 µg-1 Cmic h-1). The MicroGC was again used and data recorded automatically.*
*The potentially leachable C and N were estimated by quantifying soluble organic C (DOC) and total soluble N (TDN) in the soils at the end of the experiment. DOC and TDN were measured using a method adapted from Jones and Willett65 with a TOC analyzer equipped with a supplementary module for N (CSH E200V, Shimadzu, Kyoto, Japan). For the extraction, 30 ml of a 0.025 M K2SO4 solution were added to the soil at 70% WHC (wet soil weight equivalent to 10 g dry soil) with 5 glass balls and agitated for 30 minutes at 250 rpm at room temperature. The mixture was then centrifuged for 5 minutes at 2500 rpm at 4 °C, and the supernatant was passed through a 0.45 µm filter, which was then analyzed for DOC and TDN.*
*All measurements were done and data collected by Lauren Gillespie together with technicians.*

Timing and spatial scale

Due to the large latitudinal distribution and varying soil and climate conditions of the sites, the soil microbial communities do not necessarily have the same degree of drought history and adaptation. We therefore applied a site-specific drought treatment representative of each of the four study sites, i.e. site-specific drought intensity and duration.
We measured the CO2 and N2O fluxes before the first drought (start of the data collection), at the end of the first and second drought periods, and seven days following the rewetting of the drought exposed microcosms. The second DRW cycle started immediately after the post-rewetting flux measurement. The stop of the data collection during the experiment was the final gas flux measurement after the second rewetting period. Flux measurements were done concurrently in both the DRW and control microcosms of the same group.
As a consequence of the site-specific drought duration the DRW treatments differed in length. In order to avoid confounding experimental durations we staggered the beginning of the first drying-rewetting cycle so that all microcosms finished the two DRW cycle treatment at approximately the same time. Since it was unfeasible to stagger each microcosm individually, we regrouped the microcosms by site and by soil drying speed (i.e. drying period duration, which was determined in a preliminary test) into eight groups.
A list of all dates can be provided if needed, however this includes a minimum of 80 dates and would be better presented in a table or excel document. There are 8 start and 8 end dates. The experiment was conducted in climactic chambers and therefore not influenced by seasonal variations.

Data exclusions

Extreme data points were excluded from analysis, defined as ± 3 times the IQR of all values for each variable, to avoid biasing results by outlying points. Exact numbers and percentages of extreme data points removed per variable can be provided; this never exceeded 11% of the total number of points per group. A group was defined according to the variable: individual CO2 and N2O flux variables were grouped by tree species number, treatment, and experimental stage; cumulative CO2 and N2O fluxes, DOC, TDN, and qCO2 variables were grouped by tree species number and treatment; and the CO2 and N2O resistance and recovery indices were grouped by tree species number. Ungrouped, the number of removed values never exceeded 8% of the total values.

Reproducibility

The data presented here is tied to specific spatial and temporal ecological conditions (e.g. forest drought history, tree species presence, microbial community composition, soil property heterogeneity) which are susceptible to change. This makes exact study replication challenging and underlines the importance of including a wide range of conditions (e.g. multiple forest types, tree species, tree species combinations, climatic conditions, soil types) as done here in order to explore general, potentially reproducible, trends oppose to site-specific trends.

Randomization

*We used a separate growth chamber for each of the replicates and treatments (i.e. six growth chambers), which were included in the*

| | |
|---|---|
| Randomization | *statistical analyses as a random factor. Within each chamber, the microcosms were randomly distributed on a single shelf and re-randomized weekly.* |
| Blinding | *Blinding was not relevant to our study.* |

Did the study involve field work? ☒ Yes ☐ No

## Field work, collection and transport

| | |
|---|---|
| Field conditions | *The sites used in this study are part of a permanent network of existing mature forest plots across Europe established in 2011-2012. We included four sites ranging over a large climatic gradient: North Karelia, Finland (MAT 2.1°C; MAP 700 mm); Białowieża, Poland (MAT 6.9°C; MAP 627 mm); Râşca, Romania (MAT 6.8°C; MAP 800 mm); and Colline Metallifere, Italy (MAT 13°C; MAP 850 mm).* |
| Location | North Karelia, Finland (latitude, 62.6°, longitude 29.9°); Białowieża, Poland (52.7, 23.9°); Râşca, Romania (47.3°, 26.0°); and Colline Metallifere, Italy (43.2°, 11.2°), which correspond to typical boreal forests, hemiboreal mixed broadleaved-coniferous, mountainous mixed beech, and Mediterranean thermophilous, respectively.<br>Within each site we selected 30 m x 30 m forest plots dominated by either one tree species (mono-specific plot) or three tree species (mixed plot): North Karelia (6 mono-specific plots, 3 mixed plots); Białowieża (6 mono-specific plots, 14 mixed plots); Râşca (8 mono-specific plots, 8 mixed plots); and Colline Metallifere (10 mono-specific plots, 9 mixed plots). More details for each plot (e.g. latitude/longitude, elevation, topography) are provided in Supplementary Data file 1. |
| Access and import/export | *All plots were accessible by car/walking and we were accompanied by local scientists or guides (hired by the local site management responsible for the permanent plots). Soil samples were all collected within Europe and no permits were necessary for transportation.* |
| Disturbance | *When accessing the plots, effort was made to minimizee disturbance by following trails and avoiding unnecessary exploration. Within the plots, forest floor litter disturbance was unavoidable, but soil core holes were closed after sampling and litter replaced over the sampled area.* |

# Reporting for specific materials, systems and methods

We require information from authors about some types of materials, experimental systems and methods used in many studies. Here, indicate whether each material, system or method listed is relevant to your study. If you are not sure if a list item applies to your research, read the appropriate section before selecting a response.

### Materials & experimental systems

| n/a | Involved in the study | |
|---|---|---|
| ☒ | ☐ | Antibodies |
| ☒ | ☐ | Eukaryotic cell lines |
| ☒ | ☐ | Palaeontology |
| ☐ | ☒ | Animals and other organisms |
| ☒ | ☐ | Human research participants |
| ☒ | ☐ | Clinical data |

### Methods

| n/a | Involved in the study | |
|---|---|---|
| ☒ | ☐ | ChIP-seq |
| ☒ | ☐ | Flow cytometry |
| ☒ | ☐ | MRI-based neuroimaging |

## Antibodies

| | |
|---|---|
| Antibodies used | *Not applicable* |
| Validation | *Not applicable* |

## Eukaryotic cell lines

Policy information about cell lines

| | |
|---|---|
| Cell line source(s) | *State the source of each cell line used.* |
| Authentication | *Not applicable* |
| Mycoplasma contamination | *Not applicable* |
| Commonly misidentified lines<br>(See ICLAC register) | *Not applicable* |

## Palaeontology

Specimen provenance: *Not applicable*

Specimen deposition: *Not applicable*

Dating methods: *Not applicable*

☐ Tick this box to confirm that the raw and calibrated dates are available in the paper or in Supplementary Information.

## Animals and other organisms

Policy information about studies involving animals; ARRIVE guidelines recommended for reporting animal research

Laboratory animals: *This study did not involve laboratory animals.*

Wild animals: *Wild animals were not used in this study.*

Field-collected samples: *Field-collected soil samples were air-dried immediately after sampling and stored in airtight plastic bags for transportation and storage at room temperature (21°C) in the dark until the experiment. Before the start of the experiment, the soil samples were rewetted and the soil microbial community was left to reactivate for three weeks. After the experiment, the soil was used for post-experiment analyses (i.e. microbial stress and potential nutrient leaching).*

Ethics oversight: *No ethical approval or guidance was required. We did not work with dangerous nor foreign materials (i.e. exotic species, pathogens, etc.).*

Note that full information on the approval of the study protocol must also be provided in the manuscript.

## Human research participants

Policy information about studies involving human research participants

Population characteristics: *Not applicable*

Recruitment: *Not applicable*

Ethics oversight: *Not applicable*

Note that full information on the approval of the study protocol must also be provided in the manuscript.

## Clinical data

Policy information about clinical studies

All manuscripts should comply with the ICMJE guidelines for publication of clinical research and a completed CONSORT checklist must be included with all submissions.

Clinical trial registration: *Not applicable*

Study protocol: *Not applicable*

Data collection: *Not applicable*

Outcomes: *Not applicable*

## ChIP-seq

### Data deposition

☐ Confirm that both raw and final processed data have been deposited in a public database such as GEO.

☐ Confirm that you have deposited or provided access to graph files (e.g. BED files) for the called peaks.

Data access links
*May remain private before publication.*: *Not applicable*

Files in database submission: *Not applicable*

Genome browser session (e.g. UCSC): *Not applicable*

### Methodology

| | |
|---|---|
| Replicates | *Not applicable* |
| Sequencing depth | *Not applicable* |
| Antibodies | *Not applicable* |
| Peak calling parameters | *Not applicable* |
| Data quality | *Not applicable* |
| Software | *Not applicable* |

## Flow Cytometry

### Plots

Confirm that:

☐ The axis labels state the marker and fluorochrome used (e.g. CD4-FITC).

☐ The axis scales are clearly visible. Include numbers along axes only for bottom left plot of group (a 'group' is an analysis of identical markers).

☐ All plots are contour plots with outliers or pseudocolor plots.

☐ A numerical value for number of cells or percentage (with statistics) is provided.

### Methodology

| | |
|---|---|
| Sample preparation | *Not applicable* |
| Instrument | *Not applicable* |
| Software | *Not applicable* |
| Cell population abundance | *Not applicable* |
| Gating strategy | *Not applicable* |

☐ Tick this box to confirm that a figure exemplifying the gating strategy is provided in the Supplementary Information.

## Magnetic resonance imaging

### Experimental design

| | |
|---|---|
| Design type | *Not applicable* |
| Design specifications | *Not applicable* |
| Behavioral performance measures | *Not applicable* |

### Acquisition

| | |
|---|---|
| Imaging type(s) | *Not applicable* |
| Field strength | *Not applicable* |
| Sequence & imaging parameters | *Not applicable* |
| Area of acquisition | *Not applicable* |

Diffusion MRI ☐ Used ☒ Not used

### Preprocessing

| | |
|---|---|
| Preprocessing software | *Not applicable* |
| Normalization | *Not applicable* |

| | |
|---|---|
| Normalization template | *Not applicable* |
| Noise and artifact removal | *Not applicable* |
| Volume censoring | *Not applicable* |

## Statistical modeling & inference

| | |
|---|---|
| Model type and settings | *Not applicable* |
| Effect(s) tested | *Not applicable* |

Specify type of analysis: ☐ Whole brain ☐ ROI-based ☐ Both

| | |
|---|---|
| Statistic type for inference (See Eklund et al. 2016) | *Not applicable* |
| Correction | *Not applicable* |

## Models & analysis

| n/a | Involved in the study |
|---|---|
| ☒ | ☐ Functional and/or effective connectivity |
| ☒ | ☐ Graph analysis |
| ☒ | ☐ Multivariate modeling or predictive analysis |

| | |
|---|---|
| Functional and/or effective connectivity | *Not applicable* |
| Graph analysis | *Not applicable* |
| Multivariate modeling and predictive analysis | *Not applicable* |
